# Seroepidemiology of *Toxoplasma gondii* infection in women of child-bearing age in central Ethiopia

**DOI:** 10.1186/1471-2334-13-101

**Published:** 2013-02-26

**Authors:** Endrias Zewdu Gebremedhin, Anteneh Hailu Abebe, Tesfaye Sisay Tessema, Kassu Desta Tullu, Girmay Medhin, Maria Vitale, Vincenzo Di Marco, Eric Cox, Pierre Dorny

**Affiliations:** 1 Ambo University, Faculty of Agriculture and Veterinary Sciences, Department of Veterinary Laboratory Technology, P.O.Box 19, Ambo, Ethiopia; 2 Addis Ababa University, College of Health Sciences, School of Veterinary Medicine, Department of Microbiology, Immunology and Public Health, P.O.Box 34, Debre-Zeit, Ethiopia; 3 Addis Ababa University, College of Health Sciences, Department of Medical Laboratory Sciences, P. O. Box 9086, Addis Ababa, Ethiopia; 4 Addis Ababa University, Aklilu Lemma Institute of Pathobiology, P.O.Box. 1176, Addis Ababa, Ethiopia; 5 Italian National Reference Centre for Toxoplasmosis at Istituto Zooprofilattico Sperimentale della Sicilia A. Mirri, Palermo, Italy; 6 Gent University, Faculty of Veterinary Medicine, Salisburylaan 133, B-9820, Merelbeke, Belgium; 7 Institute of Tropical Medicine, Department of Biomedical Sciences, P. O. Box 2000, Antwerp, Belgium

**Keywords:** *Toxoplasma gondii*, Seroprevalence, Cross-sectional, Risk factors, Central Ethiopia, ELISA

## Abstract

**Background:**

*Toxoplasma gondii* infections during pregnancy can result in abortion or congenital defects. Prevalence and risk factors of toxoplasmosis in women of child-bearing age in Ethiopia are unknown. The current study was conducted with the objectives of estimating the seroprevalence and potential risk factors in acquiring *T. gondii* infection by women of child-bearing age in Central Ethiopia.

**Methods:**

A cross-sectional study was conducted from March 2011 to September 2011. Sera of 425 women were analyzed by indirect enzyme linked immunosorbent assay (ELISA). A questionnaire survey was administered for all study participants to gather information on risk factors.

**Results:**

The study revealed that anti- *T. gondii* IgG antibodies were detected in 81.4% of the samples of which 78.4% were positive for only IgG and 3.06% positive for both IgG and IgM antibodies. Seroprevalence of IgM antibodies to *T. gondii (*4.0%, 95% CI: 2.14, 5.86) was suggestive of recent infections. Of the 213 pregnant women 9 (4.2 %) were IgM reactive. Out of 17 potential risk factors investigated, univariate logistic regression showed significant association of *T. gondii* infection with study area, age, pregnancy status, raw vegetable consumption, source of water, presence of cats at home, contact with cats, HIV status and precaution during cats’ feces cleaning (P ≤ 0.05). The final logistic regression model revealed that: the probability of acquiring *T. gondii* infection by women of Debre-Zeit was 4.46 times (95% CI of adjusted odds ratio [aOR]: 1.67, 11.89; P =0.003) higher compared to women of Ambo, pregnant women were twice (95% CI aOR: 1.13, 3.59; P = 0.018) more likely to be seropositive than non-pregnant women and women who consume raw vegetable were at increased risk of infection (aOR = 2.21, 95% CI: 1.03, 4.78; P = 0.043) than women who didn’t consume.

**Conclusion:**

The seroprevalence of *T. gondii* infection in women of child-bearing age in Central Ethiopia is high. Study area, pregnancy and raw vegetable consumption are risk factors to acquire *T. gondii* infection. Educational program, antenatal screening of pregnant women and further epidemiological studies to uncover the economic and health impact of toxoplasmosis are suggested.

## Background

Toxoplasmosis is among the global major zoonotic diseases [1,2] and the third leading cause of food-related deaths in the USA [3]. It is caused by *Toxoplasma gondii*, an Apicomplexa protozoan parasite [4], with cats as the definitive host, and warm-blooded animals as intermediate hosts [5]. Humans get infections with *T. gondii* after ingesting raw or undercooked meat, by ingesting cat-shed oocysts via contaminated soil, food or water; or congenitally by transplacental transmission of tachyzoites [1,5-9]. Infection with *T. gondii* during pregnancy can result in fetal death, neonatal death or various congenital defects, such as hydrocephalus, central nervous system abnormalities and chorioretinitis [1,9,10].Toxoplasmosis is also a serious problem in immunocompromised patients [7,10]. In addition, recent studies have indicated that toxoplasmosis is a plausible risk factor for personality shifts and increased likelihood of reduced intelligence or schizophrenia [11]. Recently, highly virulent genetically atypical *strains* of *T. gondii* have been incriminated with pneumonia, even in immunocompetent individuals [12]. Serological screening for *T. gondii* antibodies should be done in women of child-bearing age as it allows identification of women at risk of acquiring infection [3] and is part of a strategic approach for prevention of congenital toxoplasmosis [13]. The seroprevalence of *T. gondii* infection among women of child-bearing age in different countries ranges from 4 to 100% [3,4]. In Africa, prevalence ranges from 25% in Burkina Faso to 75% in Sao Tome and Principe [1]. In Ethiopia, toxoplasmosis is a neglected disease and infection in women of child-bearing age is unknown. Few studies undertaken in Ethiopia reported prevalence of toxoplasmosis in the general population ranging from 20.2% to 97.7% [14-20]. With the exception of the IgG seroprevalence (20.2%, 19/94) report of Eshete *et al.*[19] no study has reported its prevalence in pregnant women in Ethiopia. Until the widespread use of antiretroviral drugs, toxoplasmosis is the most common disease complication, next to tuberculosis, among HIV seropositive admissions and deaths at Tikur Anbessa Teaching Hospital in Addis Ababa [21]. Because of the asymptomatic nature of primary *T. gondii* infection, counseling of pregnant women is of paramount importance to reduce the risk of fetal infection. Effective counseling for prevention requires knowledge of the risk factors associated with the transmission of the parasite [22]. The current study was conducted with the objectives of estimating the seroprevalence and potential risk factors in acquiring *T. gondii* infection by women of child-bearing age in Central Ethiopia.

## Methods

### Description of the study areas and population

#### Study areas

The study was conducted in Addis Ababa, Ambo, Debre-Zeit and Metehara towns of Central Ethiopia. According to CSA [23] the inhabitants of Addis Ababa city, Ambo, Ada’a-Liben and Fentale districts were 2,738,248; 110,796; 131,273 and 82,225, respectively. Addis Ababa, the capital of Ethiopia, is characterized by a biannual rainfall. Ambo is the capital of West Shoa Zone of the Oromia Regional State. Debre-Zeit and Metehara are the capitals of Ada’a-Liben and Fentale districts, respectively, and are located in East Shoa Zone within a distance of 190 kms from Addis Ababa. Fentale district has arid and semi-arid climates [24]. The location, altitude, annual mean rainfall and the mean minimum and maximum temperature of Addis Ababa, Ambo, Debre-Zeit and Metehara towns was shown in Table 1[24-26].

**Table 1:** Location, altitude, rainfall and temperature of the study areas

**Study area**	**Location**	**Altitude (masl)**	**Annual mean rainfall (mm)**	**Mean temperature (°C)**		**Ref**
	**(Longitude, altitude)**					
				**Min.**	**Max.**	
Addis Ababa	38°44^′^, to 39° 55^′^ E & 9°^′^48″N	2200 - 2500	1180.4	10.6	22.8	25
Ambo	37° 32' to 38° 3' E &	1400 - 3045	1100	13	27	26
	8° 47' to 9° 20' N					
Ada’a-Liben (Debre-Zeit)	38°38'E, & 08° 44' N	1500 - 2000	839	7.9	28	24
Fentale (Metehara)	23’ to 39°54’ E & 8° 54’ N	955 - 2007	553	29	38	24

masl = meter above sea level.

### Study population

The study populations were women of child-bearing age (pregnant and non-pregnant) aged between 15 and 49 years. Blood samples were collected from women visiting four hospitals and five health centers for antenatal follow up or medication.

### Study design

The study was cross-sectional and conducted from March 2011 to September 2011. The sample size was calculated according to Thrusfield [27] using an expected IgG prevalence of 74.4% [20], a desired precision of 0.05 with 95% level of confidence. This resulted in a sample size of 293. To take account of non-response rate and geographical clustering of the target infection the sample size was inflated by 45% to get a total sample size of 425. A total of 425 sera from 212 non-pregnant and 213 pregnant women were collected. The total sample size was distributed into the four study areas using proportional allocation (Table 2) and women were randomly selected in each study area.

**Table 2:** Patient flow and sample size distribution among selected health institutions of the study areas

**Study areas**	**Patient/women flow per year**	NareaNtotal	**Proportional sample**
Addis Ababa	639	0.35	150
Debre-Zeit	520	0.29	122
Ambo	401	0.22	93
Metehara	255	0.14	60
Total	1,815		425

### Sample collection and transportation

Blood samples (5 ml) were collected by using sterile plain Vacutainer tubes (BD Vacutainer systems, Plymouth, UK). For logistic reasons, samples were collected from nine health institutions (Addis Ababa Black line hospital, Kebena health center, Ambo hospital, Ambo health center (2), Debre-Zeit hospital, Debre-Zeit health center, Metehara hospital, Metehara health center). Blood samples were left overnight at room temperature to allow clotting and centrifuged at 3000 rpm for 10 minutes. The sera were collected in Eppendorf tubes (Eppendorf-AG, Hamburg, Germany) and stored at 4°C for 48-78 hours until transported in an ice box to the Microbiology laboratory of the School of Veterinary Medicine where they were kept at – 20°C until tested.

### Indirect IgG and IgM ELISA

Sera were analyzed for the presence of IgG and IgM antibodies against *T. gondii* by the indirect enzyme linked immunosorbent assay (ELISA) kit (Demeditec Diagnostics GmbH, Germany) conducted according to the manufacturer’s instructions. The kit has reported sensitivity and specificity of 98% and 99%, respectively. The optical densities of wells were measured by a photometer at a wavelength of 450 nm. Values higher than the cut-off (10 IU/ml) were considered positive. Values ±20% of the cut-off were equivocal but were not retested due to shortage of the kits.

### Questionnaire survey

A structured questionnaire was used to assess risk factors, which included: study area, age, residential area, house floor type, monthly income, level of education, pregnancy status, stage of pregnancy, presence of cats at home, contact with cats, precaution during cat feces handling, consumption of raw/undercooked meat, hand washing after handling raw meat, consumption of raw milk, consumption of raw vegetables, source of drinking water, exposure to soil, study participants awareness about toxoplasmosis and HIV status (predetermined). These variables were selected based on literature.

### Data management and analysis

The data were recorded in Microsoft Excel spreadsheet (Microsoft Corporation) and analyzed using STATA version 11.0 for Windows (Stata Corp. College Station, TX, USA). The seroprevalence was calculated as the number of serologically positive samples divided by the total number of samples tested. A logistic regression model was employed to assess the predictive values of the potential risk factors. Variables with more than two categories were transformed into dummy variables. The Chi-square test was used to determine associations between seropositivity and potential risk factors. The strength of the associations was assessed by odds ratios and 95% confidence intervals (CI) were calculated. Variables that showed collinearity and low frequency were not offered to final model. Results were considered significant at P ≤ 0.05.

### Ethical considerations

All study subjects were informed about the study and written informed consents were obtained from all women. Confidentiality was assured by using codes. Ethical clearance was obtained from Addis Ababa University, College of Health Sciences, School of Medical Laboratory Sciences (No. DRERC 001/11/MLS).

## Results

### Seroprevalence of toxoplasmosis

The mean age ± SD of the participants was 22.9 ± 5.21. The overall IgG and IgM *T. gondii* seropositivity were 81.4% (346/425) [95% CI: 77.70, 85.13] and 4.0% (17/425) [95% CI: 2.14 – 5.86], respectively (Table 3). The agreement in the reactivity to IgG and IgM was non-significant (kappa = - 0.1988; P-value = 1.000).

**Table 3:** Overall seroprevalence of toxoplasmosis in women of child-bearing age in Central Ethiopia

**Type of ELISA test**	**Number of women tested**	**Number positive (% prevalence)**
IgG	425	346 (81.4)
IgM	425	17 (4.0)

Seventy-eight percent (78.4%) of the women were IgG reactive and IgM non-reactive; 3.06% were both IgG and IgM reactive; 0.9% IgG non-reactive and IgM reactive and 17.6% were both IgG and IgM non-reactive (Table 4).

**Table 4:** Combined IgG and IgM anti-*T. gondii* antibodies seroprevalence in women in Central Ethiopia

**Sero reaction**	**Total**		**Pregnant**		**Non-pregnant**		**P-value**
	**(N = 425)**		**(N = 213)**		**(N = 212)**		
	**Positive**	**%**	**Positive**	**%**	**Positive**	**%**	
IgG Positive only	333	78.4	176	82.6	157	74.1	0.032
IgG and IgM Positive	13	3.06	8	3.8	5	2.4	0.403
IgG and IgM Negative	75	17.6	28	13.1	47	22.2	0.015
IgG negative and IgM positive	4	0.9	1	0.5	3	1.4	0.313
Total seropositivity	346	81.4	184	86.4	162	76.4	0.008
Total seronegativity	79	18.6	29	13.6	50	23.6	0.008

**Figure 1:**
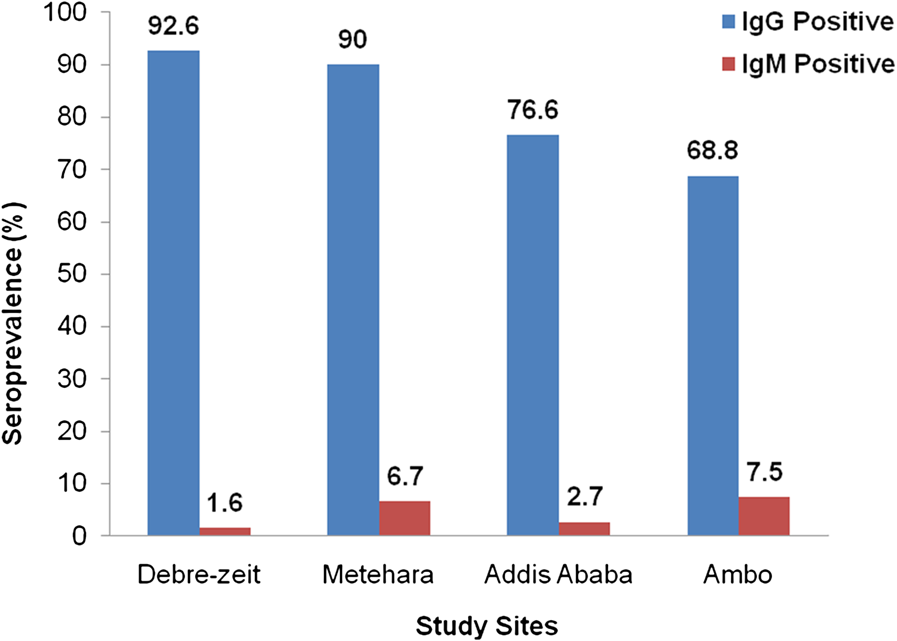
***Toxoplasma gondii *IgG and IgM seroprevalence in women of child-bearing age in Central Ethiopia.** The IgG (light blue bars) and IgM (dark red bars) percent seroprevalence were statistically significant between study sites (IgM: Chi-square = 31.706, P ≤ 0.001; IgG: Chi-square = 28.018, P ≤ 0.001).

There was significant variability in IgG and IgM seroprevalence of toxoplasmosis in different study areas **(**Figure 1).

**Figure 2:**
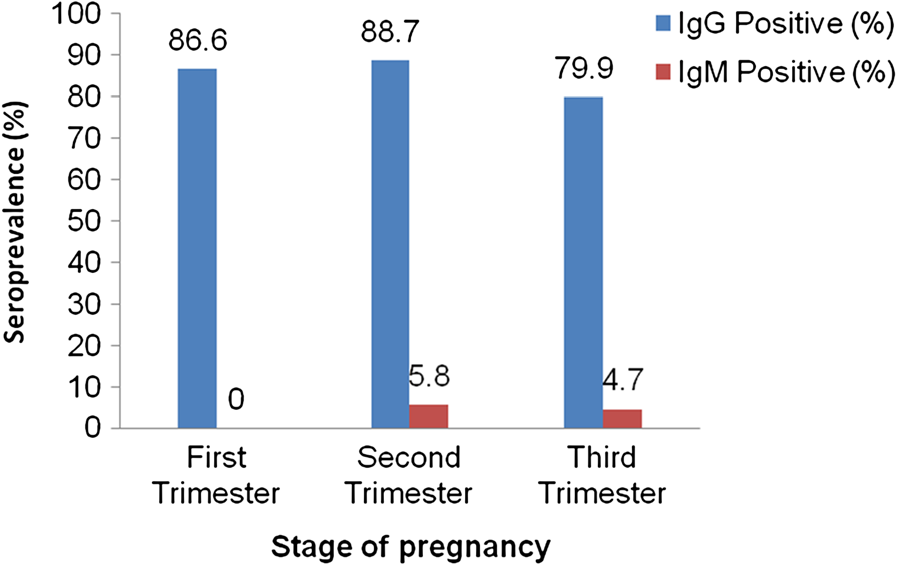
***Toxoplasma gondii *IgG and IgM seroprevalence in pregnant women of Central Ethiopia.** The figure shows difference in seroprevalence among pregnant women tested (N = 213) according to the stages of pregnancy, i.e., first trimester (conception to three months), second trimester (4 – 6 months) and third trimester (7 – 9 months). The percent seroprevalence were shown in light blue (IgG) and dark red (IgM) colored bars.

Out of 425 tested women 184 (86.4%) of the pregnant (n = 213) and 162 (76.4%) of the non-pregnant women (n = 212) were positive for anti-*T. gondii* IgG antibodies. Seventeen women (4.0%) [95% CI: 2.14, 5.86] were positive for anti- *T. gondii* IgM antibodies of which 8 (1.9%) were non-pregnant and 9 (2.1%) pregnant. Among IgM positive pregnant women, the prevalence of IgM and IgG antibodies were relatively higher in second trimester (Figure 2).

### Risk factors of *T. gondii* seropositivity

#### Univariate analysis

A comparison of epidemiological risk factors to acquire *T. gondii* between seroreactive and nonreactive women was done using logistic regression analysis. During the statistical analysis the first level of each independent variable was used as reference category. Univariate logistic regression analysis showed that study area, age, consumption of raw vegetables, source of water, presence of cats at home, contact with cats, precautions during cats’ feces cleaning, HIV status and pregnancy status were significantly associated with *T. gondii* seropositivity (P < 0.05) Table 5. Sixty percent of interviewed women keep cats at home mainly for clearing rats and mice and protect damage of stored grains. Some people also keep cats as pet animals. Univariate analysis of IgM seropositivity with pregnancy status showed that there is no significant difference between pregnant and non-pregnant women (OR: 1.07, 95% CI: 0.72, 1.61); P = 0.730) (data not shown).

**Table 5:** Logistic regression analysis of predictors of *T. gondii* infection in women of child-bearing age

**Variable**	** Category**	**IgG Positive/**	** Univariate**	**Multivariate**	
		** Total (%)**	** OR (95% CI)**	** OR (95% CI)**	** P**
Study areas	Ambo	64/93 (68.8)	reference	reference	-
	Addis Ababa	15/150 (76.7)	1.49 (0.83, 2.66)	2.42 (0.96, 6.06)	0.060
	Metehara	54/60 (90.0)	4.08 (1.58, 10.55)	2.85 (0.89, 9.16)	0.078
	Debre-Zeit	113/122 (92.6)	5.69 (2.54, 12.77)	4.46 (1.67, 11.89)	0.003
Age	15 – 20	132/180 (73.3)	reference	reference	-
(years)	21 – 25	110/130 (84.6)	2.0 (1.12, 3.57)	1.22 (0.62, 2.39)	0.566
	≥26	104/115 (90.4)	3.4 (1.70, 6.95)	1.79 (0.80, 4.00)	0.153
Residential place	Urban	244/305 (80.0)	reference		
	Rural	102/120 (85.0)	1.42 (0.80, 2.52)		
Monthly income	High	27/33 (81.8)	reference	reference	-
	Medium	28/36 (77.8)	1.29 (0.39, 4.20)	1.07 (0.29, 3.94)	0.913
	Low	291/356 (81.7)	1.00 (0.40, 2.53)	1.24 (0.43, 3.60)	0.690
Education	Tertiary	54/67 (80.6)	reference	reference	-
	Secondary	90/115 (78.3)	1.15 (0.55, 2.44)	1.58 (0.66, 3.75)	0.303
	Primary	130/162 (80.3)	1.02 (0.50, 2.10)	1.46 (0.62, 3.44)	0.389
	Illiterate	72/81 (89.0)	1.93 (0.77, 4.83)	1.93 (0.61, 6.15)	0.266
Pregnancy	No	162/212 (76.4)	reference	reference	-
	Yes	184/213 (86.4)	1.96 (1.18, 3.24)	2.01 (1.13, 3.59)	0.018
House floor	Cemented	199/252 (79.0)	reference	reference	-
	Soil	147/173 (85.0)	1.51 (0.90, 2.52)	1.25 (0.64, 2.45)	0.516
Raw meat consumption	No	204/248 (82.3)	reference	reference	-
	Yes	142/177 (80.2)	1.14 (0.70, 1.87)	1.13 (0.59, 2.18)	0.713
Hand washing after handling raw meat	Yes	319/389 (82.0)	reference	reference	-
	N0	27/36 (75.0)	1.52 (0.68, 3.37)	1.31 (0.52, 3.31)	0.573
Raw vegetable consumption	No	298/364 (81.9)	reference	reference	-
	Yes	150/171 (87.7)	2.11 (1.23, 3.64)	2.21 (1.03, 4.78)	0.043
Raw milk consumption	No	196/254 (77.2)	reference	reference	-
	Yes	48/61 (78.7)	1.22 (0.63, 2.39)	1.31 (0.59, 2.90)	0.507
Source of water	Tap	160/212 (75.5)	reference	reference	-
	Well + river	186/213 (87.3)	2.24 (1.34, 3.73)	1.99 (0.80, 4.90)	0.137
Exposure with soil	No	86/105 (81.9)	reference	reference	-
	Yes	260/320 (81.3)	1.05 (0.59, 1.85)	1.10 (0.53, 2.29)	0.795
Cat at home	No	127/167 (76.1)	reference		
	Yes	219/258 (84.9)	1.77 (1.08, 2.89)		
Contact with cat	No	130/170 (76.5)	reference	reference	-
	Yes	216/255 (84.7)	1.70 (1.04, 2.79)	1.12 (0.57, 2.21)	0.746
Precaution	Yes	120/129 (93.0)	reference		
	No	104/136 (76.5)	4.10 (1.87, 8.99)		
HIV status	Negative	247/313 (78.9)	reference	reference	-
	Positive	99/112 (88.4)	2.03 (1.07, 3.85)	1.47 (0.69, 3.14)	0.316

Key to Abbreviation: Tap = unboiled tap water; Well + river = unboiled well and river water; precaution = precaution while cat feces cleaning.

### Multivariate analysis

Multivariate logistic regression analysis showed that only study area (P = 0.003), consumption of raw vegetables (P = 0.043) and pregnancy status (P = 0.018) were independent predictors of toxoplasmosis (Table 5). The following variables were collinear: precaution during cat feces cleaning Vs contact with cats and cats at home; contact with cats Vs cats at home; house floor type Vs residential place. Thus, residential place, presence of cat at home and precaution during cleaning cats’ feces were excluded from the final model. We didn’t observe any significant interactions among the variables offered to the final model.

## Discussion

Overall, a very high seroprevalence of toxoplasmosis of 81.4% (95% CI = 77.70, 85.13) was found in women of child-bearing age in Central Ethiopia. This high figure is in agreement with other seroprevalence figures from general or selected populations in different parts of the country that ranged from 60.0 - 97.7% [14-18,20]. However, it is much higher than the 20.2% found in pregnant women referred to the National Health and Nutrition Research Center (Addis Ababa) for pregnancy related laboratory examinations [19]. The high seroprevalence could be attributed to cat density and high rate of oocyst shedding. Recently, Tiao *et al.*[28] reported that 23.9% (11/46) of the recently infected (IgM positive by ELISA) feral cats in Addis Ababa were also positive for *T. gondii* oocysts. Tiao *et al.*[28] also reported 85.4% IgG seroprevalence of *T. gondii* in 48 feral cats of Addis Ababa. Moreover, the inadequate hygiene, feeding habits and suitable climatic factors for sporulation and survival of oocysts in the environment might have additionally contributed for the high seroprevalence.

Looking into the previous prevalence reports and comparing them with the results of our study, toxoplasmosis in women is still on the rise in Ethiopia perhaps due to the lack of awareness about the disease, as indicated by all respondents in this study (unpublished observations). According to Kapperud *et al.*[29] the relative importance of the risk factors varies between countries due to differences in cultural patterns and climatic factors affecting oocyst survival. Study area, study population, sample size, age, sensitivities of serological techniques employed, cat densities in the areas and access of cat to contaminate feed and water with oocysts and geographical variability may account for some of the differences in the reported seroprevalence [4,5].

The *Toxoplasma* IgG positive and lgM negative results (78.4%, 333/425) suggest past exposure to the parasite or old infection, while positive results for IgM (4.0%, 17/425) are indicators of acute or recent exposures. Though positive IgM results are characteristic markers of recent infections further confirmation to exclude reaction of natural IgM antibody with *Toxoplasma* antigen is needed [30]. IgM antibodies can persist for a long time with no risk of congenital infections. Besides, non-pregnant women negative for IgG and IgM antibodies (47/212, 22.2%) are at risk of primary infection and should be monitored for seroconversion in case they become pregnant [30]. Among the pregnant women of the study only one (0.47%) was IgG negative and IgM positive and 8 (3.8%) were both IgG and IgM positive, which means 4.2% of the women had detectable IgM antibodies during pregnancy with potential risk of congenital toxoplasmosis warranting attention to design preventive measures.

Of the 17 potential risk factors assessed for association with *T. gondii* seropositivity, study area (P = 0.003), pregnancy status (P = 0.018) and consumption of raw vegetables (P = 0.043) were found to be independent predictors of toxoplasmosis seropositivity.

The association of the seroprevalence with the study areas may be a reflection of the life style of the people that makes them more predisposed to the infection as well as the favorable climatic conditions for *T. gondii* oocysts to sporulate. The higher seroprevalence of toxoplasmosis in women of Debre-Zeit and Addis Ababa compared to Ambo could probably be attributed to the increasing trend of raw vegetables consumption [31] and high usage of water that might have been contaminated with *T. gondii* oocysts. On the other hand, the higher seroprevalence of toxoplasmosis in Metehara compared to Ambo could be attributed to the combined effect of the presence of cats at home (91.7%), rural background of sampled women (95%), inadequate sanitation and contact with cats (93.3%) rather than raw vegetables consumption (28.3%). Furthermore, the free movement of people of Metehara leading to acquiring of the infection from other areas might additionally explain the situation. The high seroprevalence in women of Debre-zeit (92.6%) could be partly explained by the high seroprevalence reported in sheep (45.3%) [32] and goats (40.2%) [33] of same area. However, considering the arid and semi-arid nature (unfavorable for oocyst survival) and the relatively low seroprevalence of toxoplasmosis in sheep (13.4%) and goats (15.4%) of Metehara [32,33], the high seroprevalence in women of Metehara is controversial. It is possible that women might have higher exposure than animals to cat-originated oocysts since people have a close contact to pet cats. Although consumption of raw sheep and goats’ milk is a common tradition in Metehara pastoralists, only 21.7 % of interviewed women reported such a habit. We recommend further large scale study in Metehara to clarify the high seroprevalence in order to draw reliable conclusions. Variation of *T. gondii* seroprevalence with geographical location has been noted [5,7,34].

In Ethiopia, antenatal screening of toxoplasmosis is not done unless health professionals have strong suspicion of pregnancy complication in which case patients are referred to the National Health and Nutrition Research Center and other private hospitals and laboratories in Addis Ababa for a better diagnosis. Unlike the reports of Gubre-Xiaber *et al*. [20], who suggested a low risk of *T. gondii* infection during pregnancy, our findings indicated that a considerable number of pregnant women had recent infections (4.2%, 9/213 IgM positives). The current recent infection rate in pregnant women emphasizes for the need of developing antenatal care programs for toxoplasmosis in Ethiopia. In addition, the IgM and IgG seroprevalence in pregnant women indicates that they are living in a highly contaminated environment. Pregnant women are more susceptible due to immunosuppressant condition of pregnancy where the innate immunity protecting against *T. gondii* is altered during the 30^th^ and 34^th^ weeks of gestation [35]. On the other hand, Biedermann *et al*. [36] mentioned that it is difficult to associate the high seroprevalence in pregnant women with immunosuppression as reactivation of latent toxoplasmosis is rare at least in immunocompetent mothers. Although we did not perform CD^4+^ lymphocyte count of HIV positive pregnant women, there is a chance of reactivation of latent infection with a possibility of congenital transmission. Thus, strengthening the ongoing application of highly active antiretroviral therapy (HAART) and prophylactic treatment at larger scale helps considerably to prevent maternal reactivation and vertical transmission of toxoplasmosis.

*Toxoplasma gondii* infection was 2.21 times greater in individuals who ate raw vegetables than those who didn’t. The generally poor hygienic method of transport and selling of vegetables coupled with the poor quality water used to wash vegetables might have provided the opportunities for contamination by *T. gondii* oocysts. Consonant with our results, Njunda *et al.*[37] from Cameroon, Liu *et al*. [38] from China and Kapperud *et al.*[29] from Norway reported raw vegetable consumption as an important risk factor for contracting toxoplasmosis. Seroprevalence was higher in women who used unboiled river and well water for drinking purpose (87.3%) than those who used unboiled tap water (75.5%) [P = 0.002], indicating contamination of river and well waters by oocysts from felids’ feces and inadequate water management as reported by Petersen *et al*. [2]. These findings are consistent with the already documented scientific knowledge [2,37,39]. Toxoplasmosis was considered as one of water borne diseases [10,40,41].

A relative increase in the seroprevalence was observed with increasing age, as it pertains to the cumulative effect of exposure to the infective stages of the parasite. Once seroconvertion occurred, IgG antibodies persist for life. About 73.3% of the women seroconverted by the time they reach 15 - 20 years of age, most likely following acquisition of oocyst from the environment (as consumption of raw meat for this age group is uncommon). Gubre-Xiaber *et al.*[20] reported that 75% of Ethiopian children seroconvert before puberty. Several studies have indicated an increase in seroprevalence with age [3,10,13,20,34,39,42,43].

The seroprevalence of toxoplasmosis was significantly associated with the presence of domestic cats in the household (84.9%) than in their absence (76.1%) [P < 0.05]. The high seroprevalence observed in households where cats are present suggest a high environmental contamination. Felids are the only definitive hosts responsible for shedding oocysts that contaminate the environment and become infective for a long time in water or soil [5]. The present finding is in accordance with Acha and Szyfres [40] and Negash *et al.*[16] who reported strong association of seroprevalence and the presence of cats. However, Sroka *et al.*[39] and Guebre-xabier *et al.*[20] reported absence of association between seropositivity and presence of cats at home.

Raw meat is popular in Ethiopia and consumption of fresh raw beef, goat or camel meat (typically grass-fed) dipped in a spicy sauce is considered a delicacy. This began centuries ago when Bushmen couldn’t start fires to cook the meat to keep from being seen by enemies [44]. Eating raw meat (“Kurt” in Amharic language) is sort of a male thing and women just began to eat it more recently. To avoid the problems associated with *Taenia saginata*, consumption of raw goat meat [45] and sometimes mutton is nowadays practiced. Consumption of raw meat depends on culture, habit and economic condition. Despite the deep rooted tradition of raw and undercooked meat consumption and the high seroprevalence of the parasite in sheep and goats [15,31,32,46-48], no significant association was found between prevalence and raw / undercooked meat consumption (41.6% of all studied women consume raw / undercooked meat) in the current study. On the other hand, earlier studies in Ethiopia [15-18] and elsewhere [5,28] demonstrated significant association between seropositivity and behavior of raw meat consumption.

Although HIV status was not an independent predictor by the final model, HIV positive women (88.4%) are more likely to acquire *T. gondii* (OR = 2.03, 95% CI: 1.07, 3.85) in the univariate analysis, as compared to HIV negative women (78.9%). This deserves special attention as there is a high chance of reactivation of latent infection and development of toxoplasmic encephalitis [3,7].The high seroprevalence of toxoplasmosis in HIV positive women might partly be due to early child hood and teenage infection. In contrast, Biedermann *et al*. [36] and Weldemichael *et al.*[18] reported similar seroprevalence between HIV infected individuals and normal controls.

We are reporting for the first time from Ethiopia that there is a significant association between *T. gondii* infection and pregnancy, consumption of raw vegetables and use of untreated well and river water.

Committing potential errors by respondents due to recall bias and low health related knowledge (all women have no awareness about health risk of cats to humans) for some of our questions (leading to false negative response), failure to include species of food animals used for meat, failure to make further follow-up and retesting of IgM positive women and selection of sampled women who were volunteers from health institution (that might not necessarily represent the general population) are some of the limitations of our study. Hence, generalization of the results for other geographical areas or entire Ethiopian population needs to be cautiously done.

## Conclusion

In the present study we detected an overall 81.4% anti-*T. gondii* IgG and 3.96% anti-*T*. *gondii* IgM seroprevalence, indicators of latent and recent *T. gondii* infections, respectively. Our study illustrated moderately high IgM positive pregnant women (9/213, 4.2%) indicating potential for congenital transmission. We also identified study area, raw vegetables consumption and pregnancy as important risk factors to acquire *T. gondii* infection in women of child-bearing age. The high seroprevalence of *T. gondii* infection in the current study suggest the need of preventive measures, mainly education about identified risk factors, in order to reduce associated morbidities and mortalities. The results of the present study help to alert the public health delivery system of the country to undertake large scale studies and uncover the economic and health impacts and formulate guidelines and policies leading to mitigation of the potentially devastating outcomes of this zoonosis.

## Competing interest

The authors declare that they have no competing interest.

## Authors’ contribution

EZ developed the proposal, participated in the coordination and management of the study, collected and analyzed the data and drafted the article. AH participated in sample collection, laboratory testing and drafting of article with inputs from MV, V di M, EC and PD. TST, MV, V di M, EC and PD participated in the study design and edition of article. GM made contribution in the data analysis and interpretation. All authors read and approved the final manuscript.

## Pre-publication history

The pre-publication history for this paper can be accessed here:

http://www.biomedcentral.com/1471-2334/13/101/prepub
